# Anisotropic crystallite size distributions in LiFePO_4_ powders†

**DOI:** 10.1039/d1ra02102h

**Published:** 2021-04-13

**Authors:** Alexander Bobyl, Igor Kasatkin

**Affiliations:** Ioffe Institute Politekhnicheskaya ul. 26 St. Petersburg 194021 Russia bobyl@theory.ioffe.ru; St. Petersburg State University Universitetskaya nab. 7–9 St. Petersburg 199034 Russia

## Abstract

The anisotropic crystallite sizes in high-performance LiFePO_4_ powders were measured by XRD and compared with the particle sizes found by TEM image analysis. Lognormal particle size distribution functions were determined for all three main crystallographic axes. A procedure was developed to determine the fraction of the composite particles which consists of several crystallites and contains small- and large-angle boundaries. In a sample with the most anisotropic crystallites (ratio of volume-weighted mean crystallite sizes *L̄*_*V*[001]_/*L̄*_*V*[010]_ = 1.41) the number of the composite particles was at least 30%.

## Abstract 2

Large composite particles of LiFePO_4_ powders registered by TEM with at least 30% amount are recorded by XRD as smaller crystallites with at least 45% amount.

## Graphical abstract

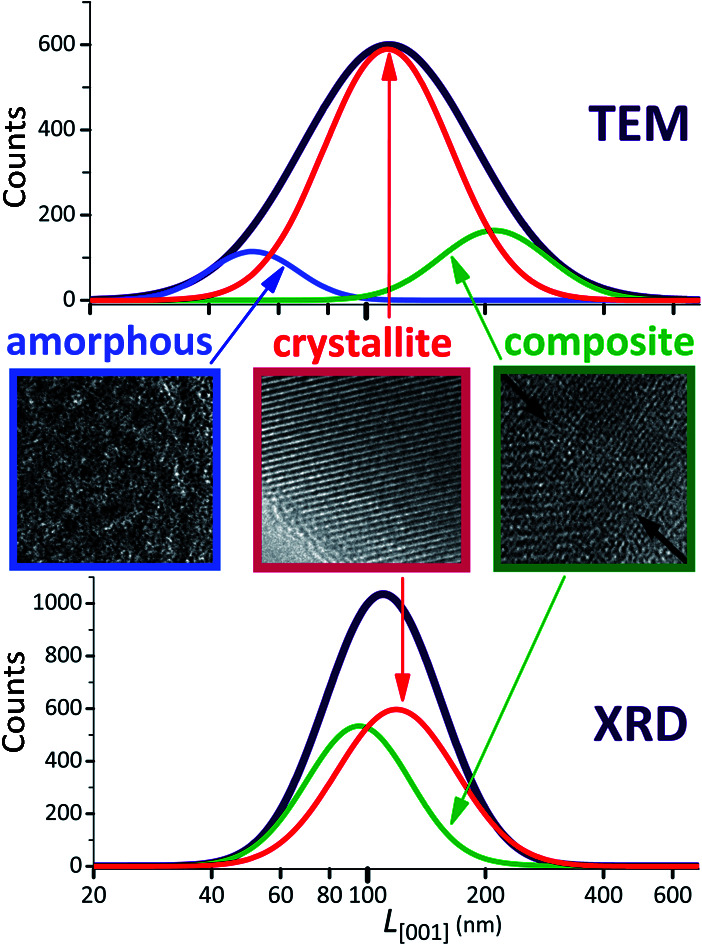

## Introduction

The efficiency of LiFePO_4_ cathodes and oxides in general can be improved by controlling the size and morphology of particles.^1–4^ A decrease in the particle size shortens the Li diffusion length and the discharge–charge time.^5,6^ This time also depends on the mechanism of the new phase nucleation on the particle surface,^7^ on the nucleation rate^8^ and on the time of the LiFePO_4_/FePO_4_ phase boundary motion in the particle. The latter depends on the structural defects in the particles: intrinsic,^9^ impurity and isovalent^1^ defects, deformations and 3D structuring of the phase boundary, and stress/deformation relationships.^10,11^ Composite particles have mechanically stable non-coherent boundaries between misoriented mosaic blocks;^12^ conglomerates are formed by ordered^13^ or chaotically disordered nanocrystallites,^14^ secondary phase particles are segregated on larger LiFePO_4_ particles.^15^ The conglomerates, even as large as 100 μm, may disintegrate and completely disappear upon additional chemically active annealing.^14^ As a rule, a mosaic microstructure negatively affects the ionic conductivity.^16^ However, a higher coefficient of diffusion along the block boundary in the particles LiCoO_2_ (ref. 17) and LiMn_2_O_4_^4,18^ was theoretically and experimentally studied.

Currently, the emerging new technologies based on computer tomographic procedures using a synchrotron^19^ or an X-ray probe^20^ allow obtaining three-dimensional (3D) images of the particle distribution in the ready-made battery electrodes. Nevertheless, the methods for determining size distributions of anisotropic particles and crystallites along their crystallographic axes remain topical. These methods include X-ray diffraction (XRD) microstructure analysis and statistical analysis of transmission electron microscopy (TEM) images. Note that XRD determines a coherent length (volume- or area-weighted mean length of the elementary columns – along certain crystallographic directions in anisotropic case, or averaged over all directions), commonly called coherent domain size or crystallite size, while TEM gives the size of particles which may consist of several crystallites. When these sizes are compared, the following problems arise:

(1) Determining size distribution functions for anisotropic crystallites, such as LiFePO_4_, on the basis of XRD has not yet become a common practice, even though it was possible in isotropic case for crystallites with high lattice symmetry.^21–25^

(2) Microscopic studies provide two sets of sizes (*L*_s_, the width, and *L*_b_, the length) measured in ensembles of differently oriented particles. A procedure is required for sorting particles in those ensembles.

(3) Presence of mosaic blocks and fused particles is obvious in some cases,^12–18,26–31^ but detection of small-angle and other boundaries separating the coherent domains requires laborious (HR)TEM studies, which can hardly be compared in statistical reliability with XRD studies.

(4) The coherently scattering domain size determined with XRD is always smaller than the particle size measured with TEM, even in a perfect crystal: each shape of a 3D crystallite predefines a certain column length distribution function. The relation between the sizes is as simple as *L*_XRD_ = 2/3*D*_TEM_ or *L*_XRD_ = 3/4*D*_TEM_ (depending on the weighting scheme) for spherical particles only. Other microstructural features and defects can complicate the situation. Generalized scheme has been developed for converting the number, surface and volume weighted particle densities.^25^

In this work we combined TEM and XRD measurements to determine the size distribution functions of anisotropic LiFePO_4_ particles and crystallites along their crystallographic axes.

### Experimental results

The following highly effective LiFePO_4_ powders were examined: no. 1, P2, Phostech Lithium;^32^ no. 2, P1, Phostech Lithium;^32^ no. 3, SPbGTI;^33^ no. 4, Golden Light Energy; no. 5, OCELL Technologies. The powders had specific capacities ranging from 145 to 170 mA h g^−1^ at 0.1C.^34,35^

### XRD studies

The methodology developed in ref. 36–38 and implemented in the MAUD software^39,40^ was used for anisotropic refinement of the crystallite size and strain values along the coordinate directions. X-Ray powder diffraction data were collected at 305 K with a Bruker D8 Discover diffractometer operating in a parallel-beam linear-focus mode at 2*θ* = 15–125°. The primary beam was conditioned with a double-bounce channel-cut Ge220 monochromator to provide CuKα_1_ radiation with a wavelength of 1.54056 Å. The specimens were prepared by dry compaction of the powders into a “zero-background” single-crystal silicon cuvette (Bruker). The data collection time was optimized to maximize StN ratio and ensure a stable refinement. The Caglioti coefficients of the instrumental profile function were refined by fitting the data for a LaB_6_ powder specimen (NIST SRM 660c) prepared and scanned under the same conditions. All of the samples were composed of phase-pure orthorhombic LiFePO_4_ with only trace amounts of impurities (<0.5%) ignored during the refinement. The refinements converged with *R*_wp_ < 9%. XRD scans of the samples are located in the ESI section.† The refinement was repeated several times from different starting conditions; the values of error reported in Table 1 (in parentheses) characterize the reproducibility. It contains the volume-averaged crystallite sizes along *L̄*_*V*[*hkl*]_ axes.

**Table 1:** Crystallite sizes along the main axes *L̄*_*V*[*hkl*]_ for the sample no. 1–5. *V̄* and *V̄*_exp_, *V̄*_cal_ – average crystallite sizes and particle volumes, respectively; *L̄*_Ss,b_ and *L̄*_Vs,b_, – surface- and volume-averaged sizes, respectively 
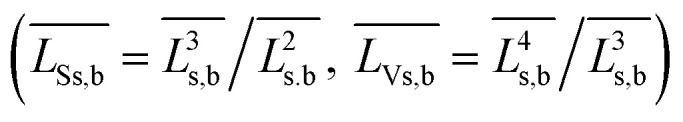
; *L̄*_Rs,b_ and *L̄*_Cs,b_ recalculated sizes from XRD studies (see Approach #2 and 3, respectively)

Sm.	*L̄*_*V*[100]_, nm	*L̄*_*V*[010]_, nm	*L̄*_*V*[001]_, nm	*V̄* = *L̄*_*V*[100]_ × *L̄*_*V*[010]_ × *L̄*_*V*[001]_ × 10^6^, nm^3^	*V̄*_exp_ = *L̄*_s_^2^ × *L̄*_b_ × 10^6^, nm^3^	*V̄*_exp_ = *L̄*_Ss_^2^ × *L̄*_Sb_ × 10^6^, nm^3^	*V̄*_exp_ = *L̄*_Vs_^2^ × *L̄*_Vb_ 10^6^, nm^3^	*V̄*_cal_ = *L̄*_Rs_^2^ × *L̄*_Rb_ × 10^6^, nm^3^	*V̄*_cal_ =*L̄*_Cs_^2^ × *L̄*_Cb_ × 10^6^, nm^3^
1	145(26)	131(13)	185(17)	3.5	1.57	4.6	6.9	2.48	3.36
2	150(10)	142(3)	158(11)	3.38	1.13	6.0	10.8	2.38	3.26
3	66(5)	82(5)	89(7)	0.49	0.70	5.7	14.4	1.86	0.45
4	230(20)	261(8)	242(30)	14.5	5.28	55	103	17.4	13.0
5	141(5)	146(15)	165(7)	3.1	1.47	5.1	8.7	2.63	3.12

### TEM studies

**Fig. 1:**
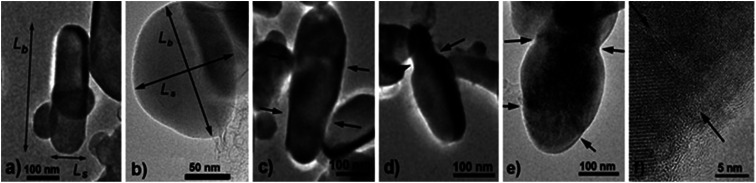
TEM images of LiFePO_4_ powders in the samples: (a) no. 1, (b) no. 2. The measured width *L*_s_ and length *L*_b_ of the particle are shown with arrows. (c)–(e), (f) – fused and mosaic particles, respectively. The arrows show the boundaries between the blocks.

**Fig. 2:**
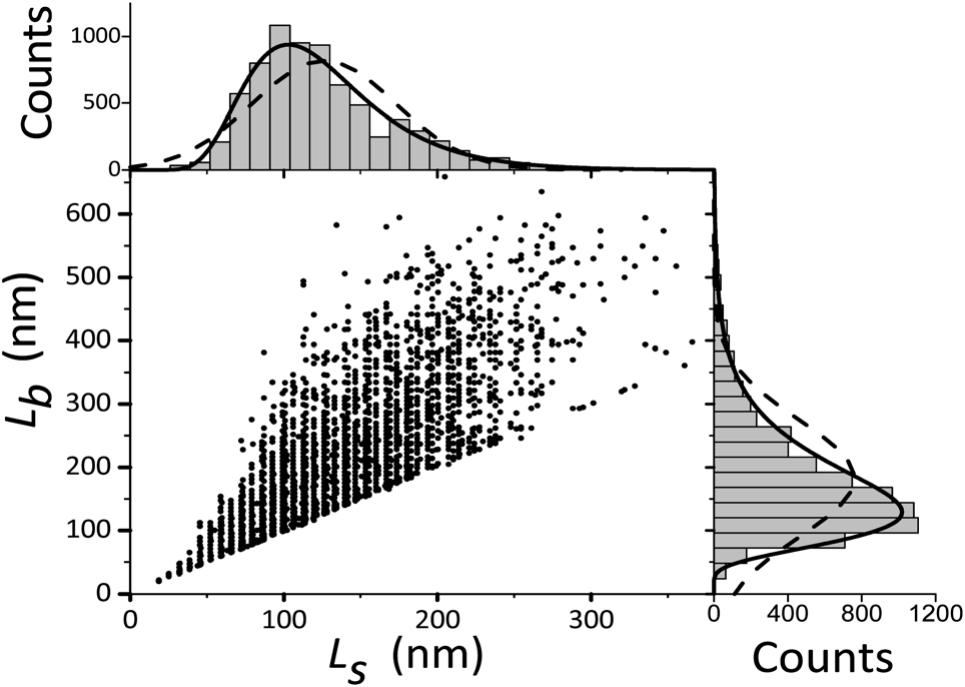
Width *L*_s_ and length *L*_b_ of LiFePO_4_ particles in sample no. 1. The corresponding frequency histograms are fitted with lognormal and Gaussian functions (solid and dashed lines, respectively).

For TEM studies, the powders were sonicated in a mixture of distilled water and ethyl alcohol (∼5–10%) for 5–10 min to separate coalescent particles. The resulting suspension was deposited on a Cu supporting grid covered with a 2–3 nm thin amorphous carbon film. The samples were examined with a JEM TEM at an accelerating voltage of 200 kV. The images were recorded with a 2048 × 2048 pixels Gatan CCD camera. In high-resolution mode the point resolution was 0.14 nm. At least 20 non-overlapping images were recorded for each sample at a magnification of 5000×. The samples contained amorphous carbon and graphene layers covering the LiFePO_4_ particles. The ordered and amorphous carbon shells were 5 nm and up to 20 nm thick, respectively. Fig. 1 shows fragments of TEM images of LiFePO_4_ powders for the samples no. 1 and 2, which demonstrated the maximum and minimum size anisotropy, respectively (see Table 1). The quantitative data (Fig. 2) used to construct the particle size distribution histograms were obtained with the Image Tool 2.0 software. The standard errors of the mean particle size were in the range of 1–3 nm.

## Analysis of experimental results

Determination of particle size distribution functions along the crystallographic axes [100], [010], [001] requires several steps of XRD and TEM data processing. For our measurements we used a series of powders with the largest differences in the mean particle sizes. First, the degrees of particle anisotropy and size variance were evaluated. The following 3 Approaches were tried.

### Approach #1

From both XRD and TEM the volume-averaged crystallite and particle sizes, respectively, can be extracted. If the fraction of mosaic particles is not large, we can assume that all particles have identical shapes, intermediate between that of a rectangular parallelepiped^13,41^ with truncated edges and of a 3-axis ellipsoid.^29^ In any case the axes are aligned with the main crystallographic directions. For simplicity, we consider them to be parallelepipeds. Then the mean volume of crystallites is easily obtained by *V̄* = *L̄*_*V*[100]_ × *L̄*_*V*[010]_ × *L̄*_*V*[001]_. In Table 1 the arithmetic *L̄*_s_^2^ × *L̄*_b_, surface- *L̄*_Ss_^2^ × *L̄*_Sb_ and volume-weighted *L̄*_Vs_^2^ × *L̄*_Vb_ mean values are listed. It was assumed that the smaller size of a particle seen in a TEM image was equal to its size along the viewing direction.

It can be seen in Table 1 that the sample no. 3 has the minimum crystallite volume of 0.49 × 10^6^ nm^3^, sample no. 1, 2, 5 have the medium values of about 3 × 10^6^ nm^3^; and the sample no. 4 has the largest volume of 14.5 × 10^6^ nm^3^. These values correlate with the arithmetic mean particle volumes. However, for comparability of TEM and XRD sizes, both should have the same weighting scheme – volume-averaged. In that case no correlation is observed. Even though this approach failed in our study, it can be applicable for particles with a plate-like and needle-like shape.^42,43^

### Approach #2

Using TEM measurements, we first calculate the volume of each particle, *V*_i_ = *L*_is_2 × *L*_ib_, and then determine the average volume *V̄*. Further, by calculating the ratio 
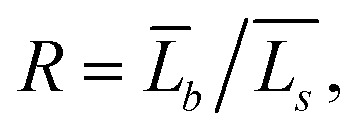
 we can determine the values of *L̄*_Rs_ and *L̄*_Rb_ as1
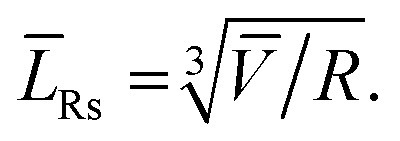


This improves the correlation, especially for the sample no. 3, as seen from Table 1. However, the volumes obtained for the rest of the samples appear smaller than the crystallite volume, which is obviously nonsense, since a coherent domain cannot be larger than a particle size.

### Approach #3

**Fig. 3:**
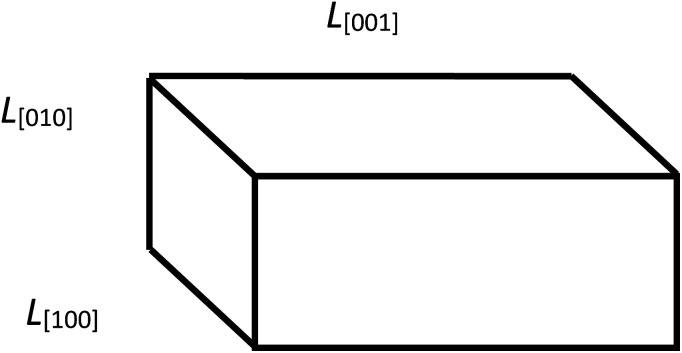
Geometric model of a particle with its edge lengths approximately corresponding to those found in the sample no.1: *L̄*_[001]_ > *L̄*_[100]_ > *L̄*_[010]_.

To determine the parameters of the particle size distribution functions in LiFePO_4_ powders along the crystallographic axes [100], [010], [001], the results of XRD measurements are used to estimate the orientation fractions in the ensembles of *L*_s_ and *L*_b_. Those fractions are further used to decompose *L*_s_ and *L*_b_ into components. We continue assuming a rectangular parallelepiped shape of particles (Fig. 3). Using the crystallite sizes measured along the main axes with XRD (Table 1) we estimate the fractions of particle orientations in the ensembles of *L*_b_ and *L*_s_ and use the recalculated values *L̄*_Cb_ and *L̄*_Cs_ (Table 1) to compare the particle volumes. To estimate *L̄*_Cb_ and *L̄*_Cs_ we assume that the probability of a crystal facet to be aligned with the object plane of the microscope is proportional to its area. For example, the normalized probability for the (100) facet is given by

2 *P*_(100)_ = *L̄*_*V*[010]_ × *L̄*_*V*[001]_/(*L̄*_*V*[010]_ × *L̄*_*V*[001]_ + *L̄*_*V*[100]_ × *L̄*_*V*[001]_ + *L̄*_*V*[100]_ × *L̄*_*V*[010]_).

In the sample no. 1 the average crystallite sizes are unequal: *L̄*_[001]_ > *L̄*_[100]_ > *L̄*_[010]_. We assume that *L̄*_Cb_ for the particle length consists of two parts: one with the size *L̄*_*V*[001]_ and the probability *P*_(100)_ + *P*_(010)_, and the other with the size *L̄*_*V*[010]_ and probability *P*_(001)_. Similarly, the *L̄*_Cs_ for the particle width consists of two parts: with the size *L̄*_*V*[100]_ and the probability *P*_(001)_ + *P*_(010)_ and with the size *L̄*_*V*[010]_ and probability *P*_(100)_. The values of *L̄*_Cb_, *L̄*_Cs_ and the relative fractions *R*_b_, *R*_s_ are further used for decomposing the experimental size distribution functions. For the sample no. 1 we can obtain the following values:

3 *L̄*_Cb_ = *L̄*_*V*[001]_(*P*_(100)_ + *P*_(010)_) + *L̄*_*V*[010]_ × *P*_(001)_ = 184.7 × 0.727 + 145.4 × 0.273 = 174.1 nm, *R*_b_ = 0.727/0.273 = 2.66

and

4 *L̄*_Cs_ = *L̄*_*V*[100]_(*P*_(001)_ +*P*_(010)_) + *L̄*_*V*[010]_ × *P*_(100)_ = 131.3 × 0.619 + 145.4 × 0.320 = 136.7 nm, *R*_s_ = 0.619/0.320 = 1.934.

It can be seen in Table 1 that the volumes calculated by using *L̄*_Cb_ and *L̄*_Cs_ are close to those calculated from the XRD measurements: the difference is within 10%. Considering the errors of XRD crystallite size determination (Table 1) this can be seen as quite an acceptable agreement. It is essential that the volume-weighted XRD sizes are used here: this ultimately accounts for the rather small error in *L̄*_Cb_ and *L̄*_Cs_. It should be kept in mind that these values are not equal to the parameters of the distribution functions shown in Fig. 2. As discussed below, they are easily calculated from the experimental distributions of *L*_b_, *L*_s_.

**Fig. 4:**
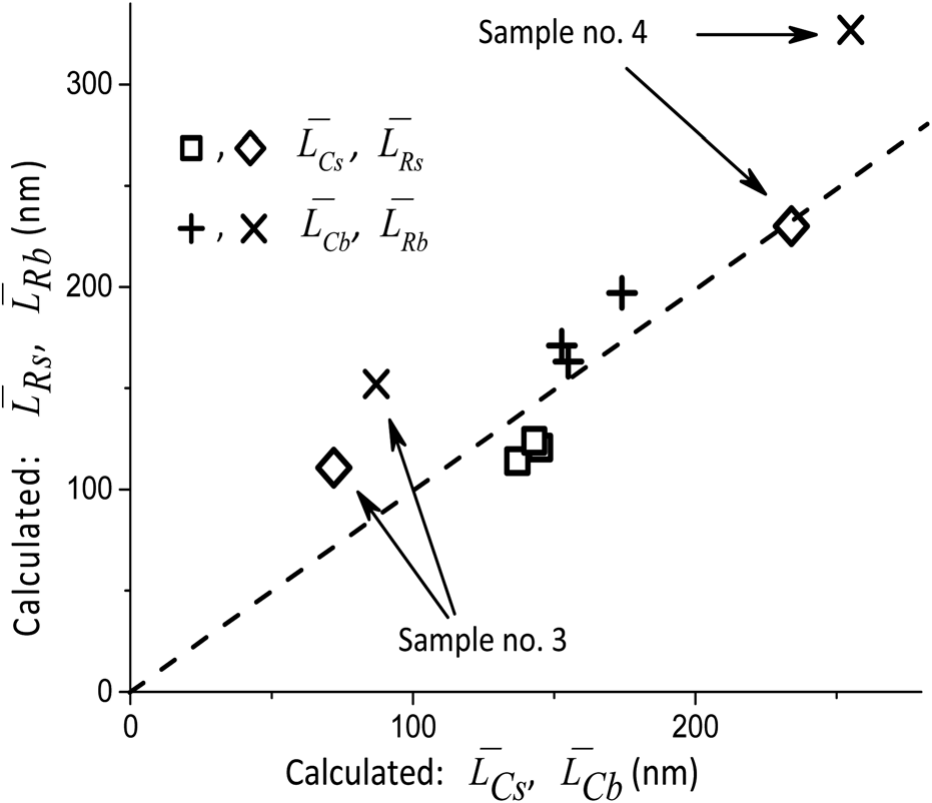
Comparison *L̄*_Rs_, *L̄*_Rb_ and *L̄*_Cb_, *L̄*_Cs_ calculated by Approaches #2 and #3, respectively. The dashed line corresponds to their equality. The sizes of the points correspond approximately to the errors in the calculations (<10%) which result from the experimental errors.

Thus, the main result of the Approach #1 is the validation of XRD measurements and simulations. Fig. 4 illustrates the results of the Approach #2. Although there are significant deviations for the samples with the minimum and maximum average particle sizes, the possibility to rapidly check the adequacy of TEM measurements is certainly useful. Finally, with the Approach #3 the results of XRD measurements are used to determine the fractions *R*_b_ and *R*_s_ of the orientations [010], [001] and [010], [100] in the distributions of *L*_b_ and *L*_s,_ respectively. Below we describe their decomposition into two components with the [010] direction being common for both. It should be noted that the types of the size distribution functions along the axes can only be obtained from TEM measurements.

## Decomposition of the experimental distributions of *L*_b_, *L*_s_ into the distributions of *L*_[100]_, *L*_[010]_, *L*_[001]_

**Fig. 5:**
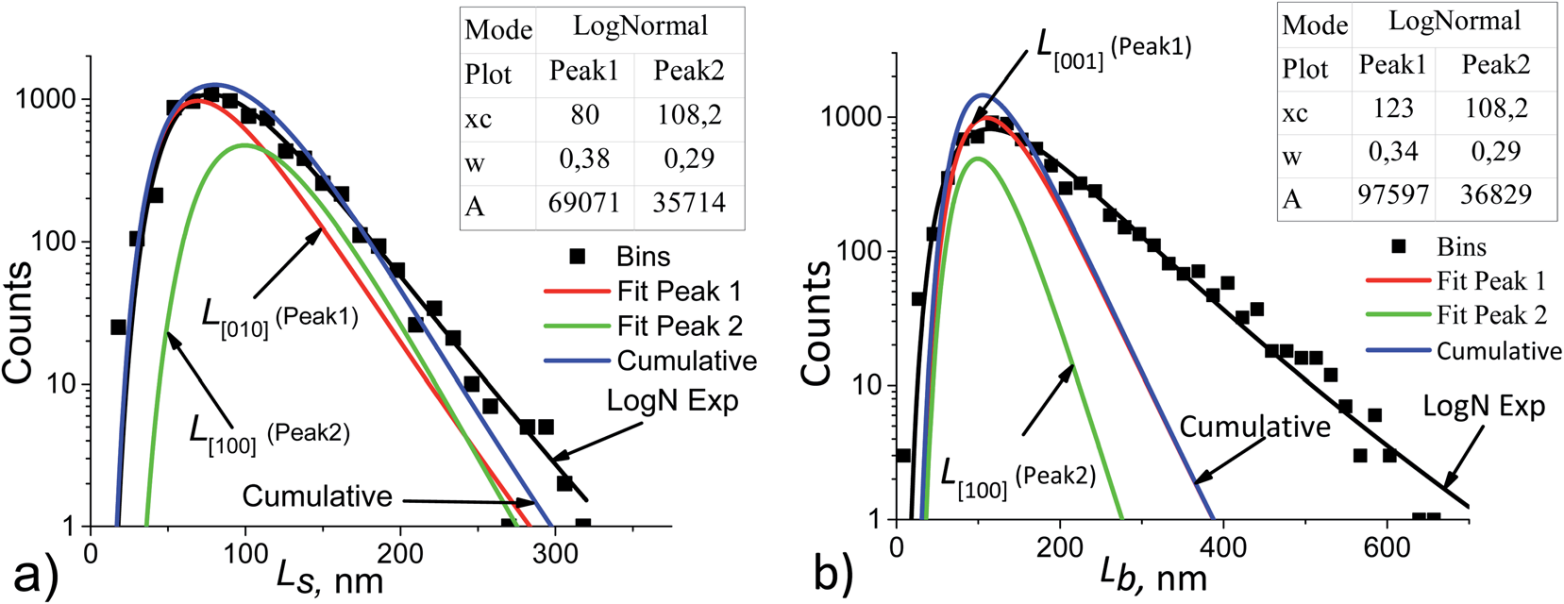
*L*_s_ (a) and *L*_b_ (b) particle size distribution histograms in sample no. 1 (black points) decomposed into the components. The basic parameters of the resulting LogNormal functions are shown in the insets using the notation of eqn (5).

Fig. 2 shows that the distribution histograms of *L*_s_ and *L*_b_ are well fitted by lognormal function with mean *xc*5
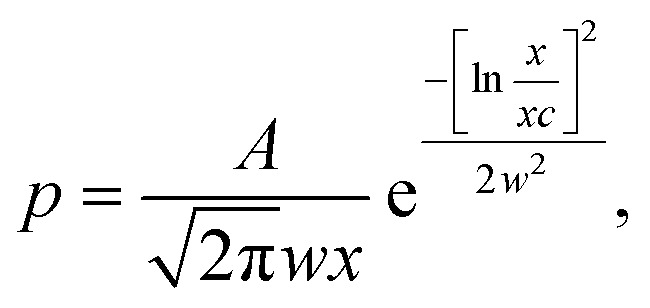
where *w* – standard deviation. According to,^44,45^ this is a consequence of critical nucleus size existence during nucleation of crystals. Fig. 5 shows the results and the parameters of the *L*_s_ and *L*_b_ distributions decomposed into the components.

To make decomposition unique, the following assumptions were taken:

(1) if the *L*_s_ and *L*_b_ distributions follow lognormal functions, then their components are also lognormal;

(2) the particle growth rate is independent of its size, but depends on the facet orientation and on the technological conditions, *e.g.*, on the stock composition.^42^ This allows using the averaged values of the fractions *R*_s_ and *R*_b_ for all points of the *L*_s_ and *L*_b_ distributions, respectively. For example, in Fig. 5a the following equation is satisfied:

6 *R*_s_ = *A*_1_/*A*_2_ = 69071/35714 = 1.934,

which corresponds to the value given above in eqn (4);

(3) the coherent domain size is strictly smaller than the particle size due to the possible existence of a mosaic substructure, coalescence of crystallites, internal boundaries with or without amorphous layers. Therefore, the cumulative curves (sums of the components) may not coincide with the functions which approximate the experimental histograms (log *N* exp in Fig. 5);

(4) the volume-averaged sizes *L̄*_*V*_ can be calculated using *L*_[*hkl*]_ obtained from the decomposition of the TEM size distributions *L*_s_ и *L*_b_. According to,^22–24^ the volume-averaged size is equal to the ratio of the fourth and the third moments of the distribution function of the linear (observed) size *L*7
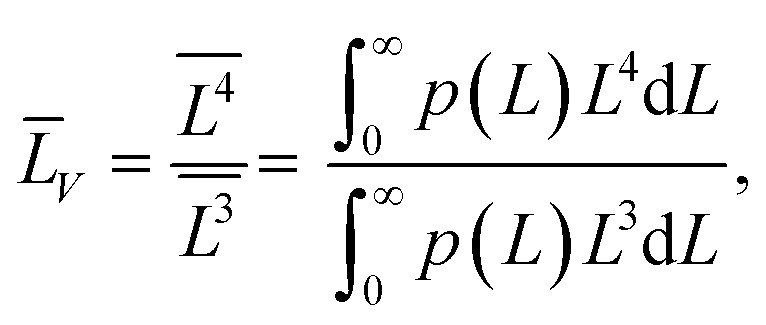
where *p*(*L*) is the size distribution function (eqn (5)). If the distribution function of *N* particles is set by the histogram *p*_*i*_(*L*_*i*_), then the integration is replaced by summation^23^8
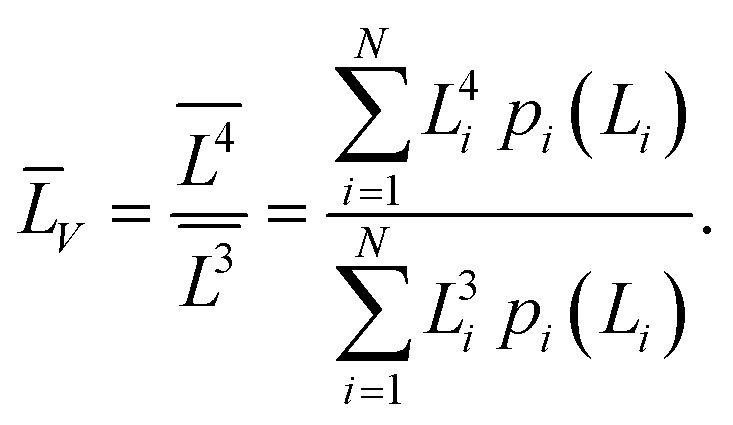


Table 2 compares the calculation results with of XRD measurements. The similarity in the sizes indicates a satisfactory decomposition;

**Table 2:** Comparison of the averaged sizes obtained from the decomposition of TEM distributions with the results of XRD measurements

	*L*_b_	Decomposition *L*_b_		*L*_s_	Decomposition *L*_s_	
		Peak 1, *L*_[001]_	Peak2, *L*_[010]_		Peak1, *L*_[010]_	Peak2, *L*_[100]_
∑*L*_b_, ∑*L*_s_, ∑*L*_[*hkl*]_, nm	1235500	734193	237773	719260	446604	285178
*L̄*_b_, *L̄*_s_, *L̄*_[*hkl*]_, nm	167.0	130.3	112.6	97.2	86.9	111.6
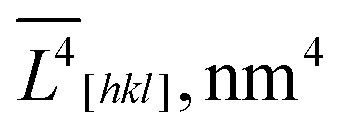		5.7664 × 10^8^	2.6617 × 10^8^		1.2787 × 10^8^	6.5295 × 10^11^
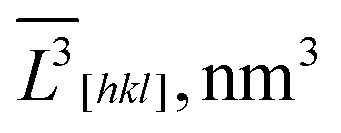		3.1290 × 10^6^	1.8365 × 10^6^		0.9746 × 10^6^	1.7839 × 10^6^
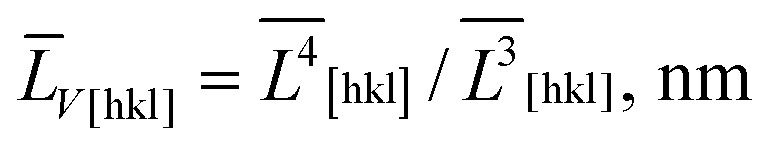		184.29	144.93		131.20	143.20

**XRD sizes**						
*L̄*_*V*[*hkl*]_, nm		184.7	145.4		131.3	145.4

**Fig. 6:**
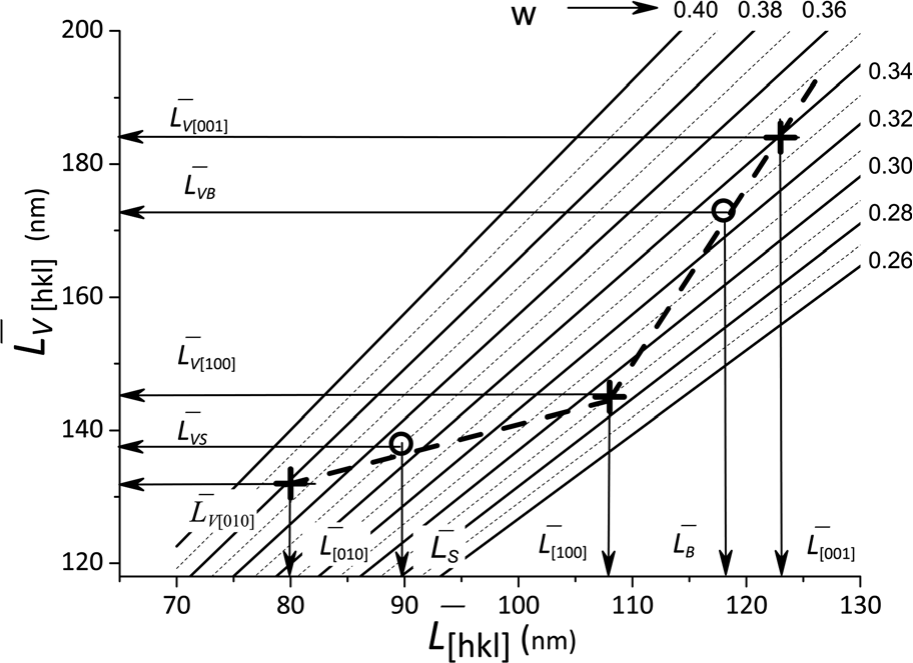
Lognormal mean sizes *L̄*_[*hkl*]_ obtained by decomposing the TEM histograms into components and the volume-averaged sizes *L̄*_*V*[*hkl*]_ obtained directly from the XRD measurements. The family of straight lines is plotted for different *w* values (standard deviation of the lognormal function) in eqn (9).

**Fig. 7:**
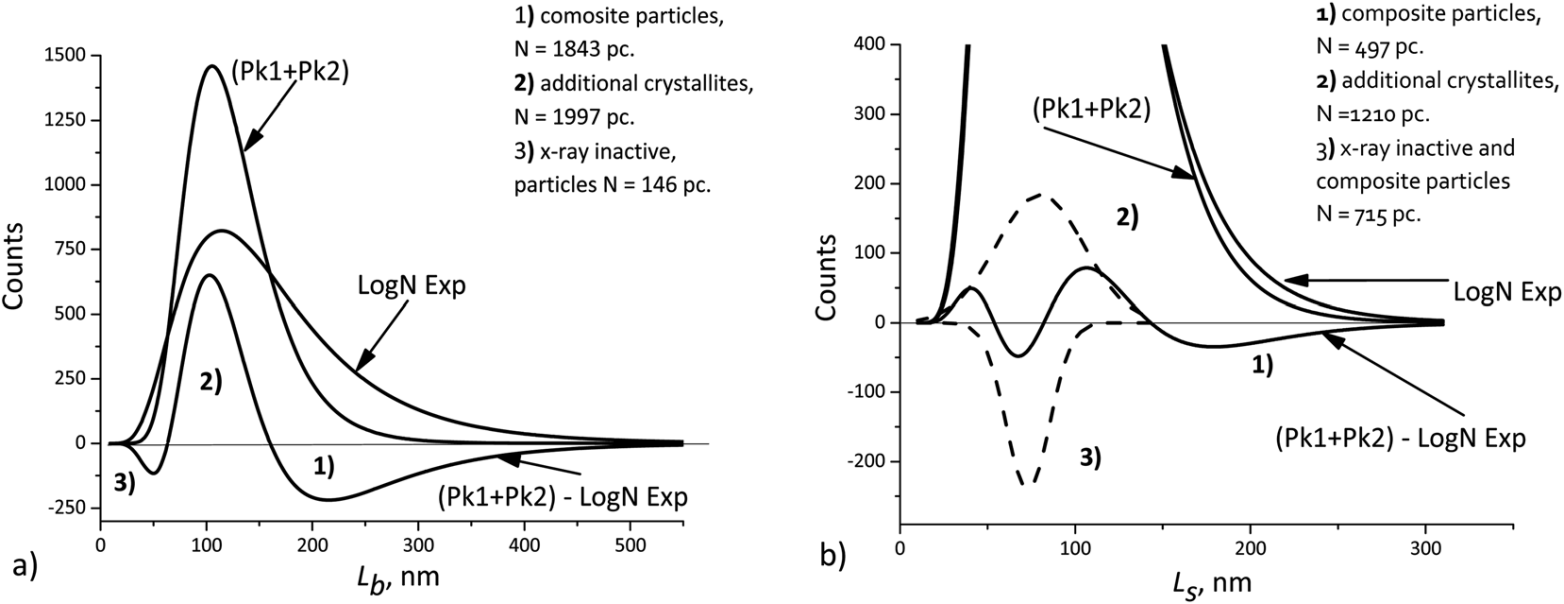
Deviations of the cumulative curve from the experimental ones (LogN Exp): (a) *L*_b_, (b) *L*_s_. The dashed curves – decomposition of the difference curve.

(5) for the component *L̄*_*V*[100]_, the LogN function parameters obtained from decomposition of *L*_s_ and *L*_b_, should be the same. This is seen from the comparison of Fig. 5a and b. Uniqueness of the decomposition into lognormal components can be checked as follows. From the properties of lognormal function^30^ it follows that9
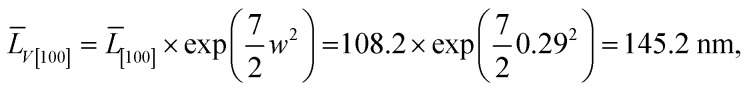
where the numerical values correspond to the “Peak 2” in Fig. 5a. All of the mean sizes obtained for the crystallites, *L̄*_[*hkl*]_ and the volume-averaged values *L̄*_*V*[*hkl*]_ are collected in Fig. 6, which also shows a parametric family of the curves for the eqn (8). With the circles, the calculation results for the cumulative distributions are shown. It can be seen that they are closer to the larger contribution in accordance with the values of *R*_s_, *R*_b_;

(6) boundaries should preferably subdivide particles into mosaic blocks along the [001] direction, since the size *L̄*_[001]_ is larger than *L̄*_[010]_, *L̄*_[100]_. This explains a larger deviation of the cumulative curve from the experimental one for large particles in the decomposition of *L*_b_, as seen in Fig. 6. However, Table 2 also shows significant deviations for *L*_s_. A detailed quantitative analysis is shown in Fig. 7. Three regions and types of particles are identified: those with a mosaic substructure; with additional crystallites and X-ray-inactive ones, only observed in TEM.

Theoretical studies demonstrated that the mosaic block sizes change in LiFePO_4_ and FePO_4_ during cycling due to the motion of edge dislocations,^26^ and the energy of a boundary depends on the degree of its coherence^27^ and lithium vacancy fraction.^4^ A special case is represented by coherent boundaries with superstructures.^28^ Highly symmetric coherent twin boundaries were found in LiCoO_2_ (ref. 17), and it was shown that the energies of Li diffusion along and across the boundary were 0.2 eV and 0.4 eV, respectively. Degradation of the LiCoO_2_ particles associated with the appearance of voids and cracks at the twin boundaries was studied in details in ref. 46.

Finally, we should keep in mind that the accuracy of XRD domain size measurements can typically be limited by anything but the number of particles. At the same time, TEM measurements, even those performed with the use image processing software, seldom involve more than 10^4^ particles. Nevertheless, such a number can be sufficient, because the errors of both methods become comparable in magnitude. This allows detecting small differences, such as those shown in Fig. 7, when the average crystallite sizes obtained from XRD measurements are compared with the sizes obtained from TEM measurements. The interpretation is based on the assumptions about the possible mosaic substructure of particles, and the quantities indicated in Fig. 7 are statistically significant.

## Conclusions

XRD and TEM data were combined to obtain the size distribution functions of LiFePO_4_ particles along the [100], [010] and [001] crystallographic directions.

Information on the anisotropy of size-distribution functions can be used to analyze the relations between the battery capacity and the charge–discharge rate.^5,6^ The fraction of composite (fused) particles consisting of several crystallites can be used to estimate the ion diffusion length along the block boundaries; the activation energy of such diffusion may differ significantly from the bulk values.^4,16,27,47^

The frequency distribution functions of different particle dimensions *L*_s_ and *L*_b_ can be decomposed into the components *L*_[*hkl*]_ by careful accounting for the anisotropy of crystallites extracted from XRD measurements.

The cumulative *L*_[*hkl*]_ curves obtained by summation of the components do not coincide with the experimental curves. The difference between these curves (Fig. 7) can be used to estimate quantitatively the percentage of mosaic particles. In our case large composite particles of LiFePO_4_ powders registered by TEM with at least 30% amount are recorded by XRD as smaller crystallites with at least 45% amount.

Possible ways of using the obtained results are described in the ESI section,† available from the article site or from the author.

## Conflicts of interest

There are no conflicts to declare.

## Supplementary Material

RA-011-D1RA02102H-s001
