# Supplementary material for: Anisotropic crystallite size distributions in LiFePO4 powders
Source: RSC Adv. 2021 Apr 13;11(23):13799–805. doi: 10.1039/d1ra02102h (PMC8697584; doi:10.1039/d1ra02102h)
Supplement: RA-011-D1RA02102H-s001 [file RA-011-D1RA02102H-s001.pdf]

Electronic Supplementary Material (ESI) for RSC Advances.
This journal is © The Royal Society of Chemistry 2021

Supporting Information
for *RSC Advances.*

Anisotropic crystallite size distributions in $LiFePO_4$ powders
Alexander Bobyl, Igor Kasatkin

Fragments of XRD scans.

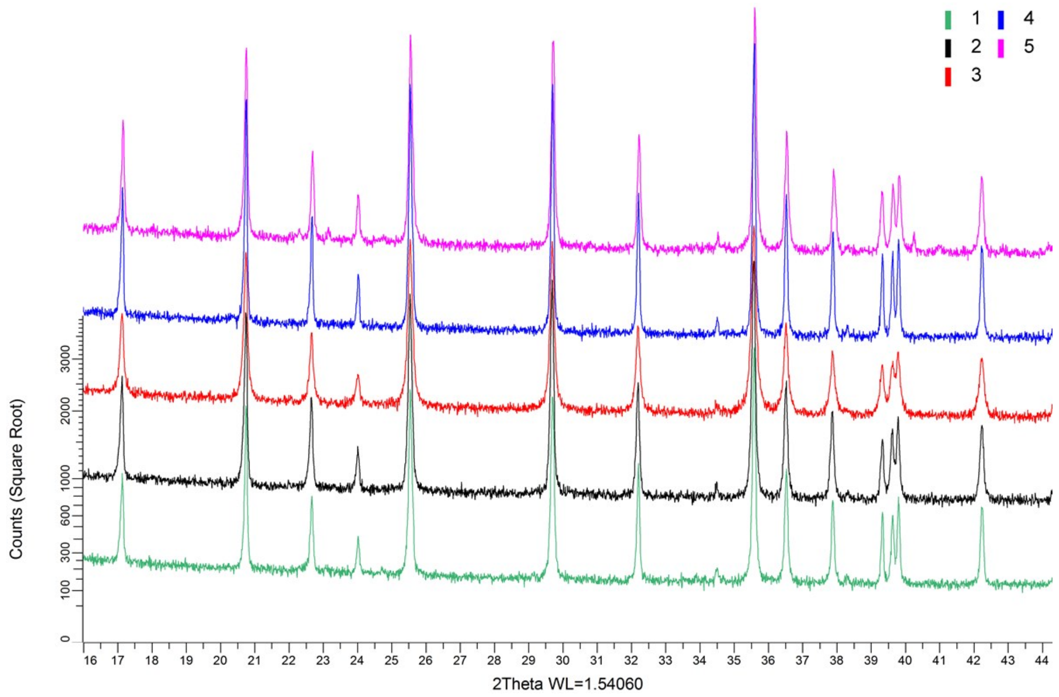

Figure S1. Fragments of XRD scans of the samples studied in this work.

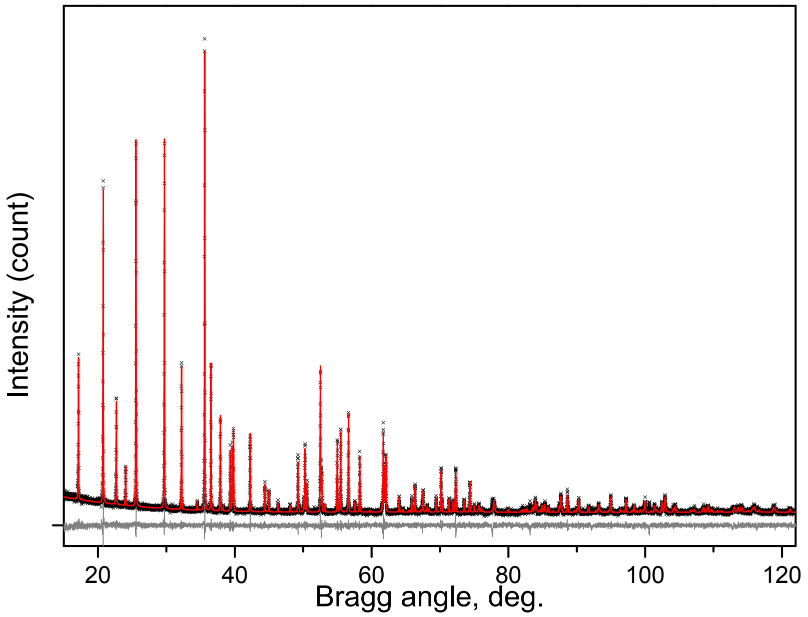

Figure S2. Typical Rietveld plot obtained for a $LiFePO_4$ sample (# 4) after refinement with MAUD software (R_{wp} = 8.5%). Black asterisks – experimental data; red line – calculated profile; grey line – difference curve.

About reproducibility. We addressed the problem of reproducibility by multiple times fitting the same datasets, each time starting from different model parameters, as mentioned in Section 2.1. It is the reproducibility that the errors reported in Table 1 characterize.
We tried different Rietveld programs to analyze our data, and have finally chosen MAUD because it treated anisotropic crystallite sizes in the most straight-forward way based on the Popa model. With TOPAS (5.0), we used the AnisoCS plugin written by Ectors et al. [1] It treated the crystallite shape as an ellipsoid, but gave unsatisfactory results. From a discussion with the author of the plugin we realized that this could be a consequence of a wide size distribution. MAUD did not have such a problem.
In general, TOPAS and MAUD produced very similar crystallite volumes in isotropic and anisotropic mode, respectively.

About microstrain. We checked that microstrain did not play any significant role in our samples. The values of maximum microstrain never exceeded 3E-4 (as determined by TOPAS) and could safely be ignored. In MAUD refinements we assumed zero microstrain.

About impurity phases. According to the standards of XRD phase analysis, the samples studied in our work should be characterized as containing no impurity phases above the detection limit. We tried to jump a bit above the standards: that is why some unavoidable and unidentified impurities were detected (below 0.5%) and mentioned in the paper. In Rietveld refinements the experimental intensities of the peaks were in good agreement with those calculated from a single-phase model, even though the impurity phase(s) were ignored.

About spherical shape. We don't assume a spherical shape of the particles. Such a shape was only mentioned in the Introduction section as an example of an oversimplified model.
Our choice of a prism (parallelepiped, cuboid, etc) as a typical particle shape was the simplest starting assumption possible. Such a choice also implies that we used no model of column length distribution function convoluted with the particle size distribution function. In our future work we shall certainly come to more complicated models.

2D and 3D distribution

2D Normal distribution is described by the equation

$$f(x_1,x_2) = \frac{1}{2\pi\sigma_1\sigma_2\sqrt{1-\rho^2}} exp\left\{ -\frac{1}{2(1-\rho^2)}\left[\left(\frac{x_1-m_1}{\sigma_1}\right)^2 - 2\rho\left(\frac{x_1-m_1}{\sigma_1}\right)\left(\frac{x_2-m_2}{\sigma_2}\right) + \left(\frac{x_2-m_2}{\sigma_2}\right)^2\right]\right\},$$

(S1)

where $m_{1,2}$, $\sigma_{1,2}$ and ρ– mean, variance and covariance, respectively.[1]

3D Normal distribution is described more compactly by the matrix equation

$$f(\bar{x}) = \frac{1}{\sqrt{(2\pi)^3 detK}} \exp\left[-\frac{1}{2}(\bar{x}-\bar{m})^T K^{-1}(\bar{x}-\bar{m})\right], \quad (S2)$$

where $\bar{x} = \begin{bmatrix} x_1 \\ x_2 \\ x_3 \end{bmatrix}$, mean $\bar{m} = \begin{bmatrix} m_1 \\ m_2 \\ m_3 \end{bmatrix}$, correlation moment matrix

$K = \begin{bmatrix} \sigma_1^2 & r_{12}\sigma_1\sigma_2 & r_{13}\sigma_1\sigma_3 \\ r_{21}\sigma_2\sigma_1 & \sigma_2^2 & r_{23}\sigma_2\sigma_3 \\ r_{31}\sigma_3\sigma_1 & r_{32}\sigma_3\sigma_2 & \sigma_3^2 \end{bmatrix}$, σ_i -variance , r_{ik} – covariance. For Lognorm $\bar{x} = \begin{bmatrix} lnx_1 \\ lnx_2 \\ lnx_3 \end{bmatrix}$.

From equation (S2), one can obtain three projections on the corresponding axes (marginal distributions)

$$f(x_i) = \frac{1}{\sqrt{2\pi}\sigma_i} exp\left[- \frac{(x_i - m_i)^2}{2\sigma_i^2} \right] \quad (S3),$$

which do not contain any covariances.

In our **Approach # 3** the results of TEM measurement approximations by marginal distributions (3), the definition of De Brouckere's diameter (volume-weighted mean diameter) are used

$$D_i[4,3] = \frac{\sum_{k=1}^{N} x_{ik}{}^4 f_{ik}}{\sum_{k=1}^{N} x_{ik}{}^3 f_{ik}} = l_{Vi} \quad , \quad (S4)$$

The last equality means that $D_i[4,3]$ are taken to be equal to the X-ray measurements of anisotropic l_{Vi} . The reason for this is specific features of X-ray scattering on volume columns oriented along the crystallographic axes and their intensity averaging over the ensemble of powder crystallites.

For 1D ion diffusion, to describe the dependence of the electrochemical experimental parameters of powders on the crystallite size, it is sufficient to use the marginal distributions (S3). But to describe 2D processes, information about the covariance is needed.[1] Consider the case of lamellar crystallites with a rectangular parallelepiped shape, the thickness of which x_3 is much less than the length and width, $x_1 > x_2 >> x_3$. From the values of the initial experimental results of TEM measurements, the covariance value ρ can be determined. The results can be represented in the form of square matrix NxN sizes with the number of samples $f_{i,k}$ fall in cell coordinates i,k. As an example, **Figure 4S** shows a possible variant of such matrix isoline and two histograms G_1 and G_2 of marginal distributions.

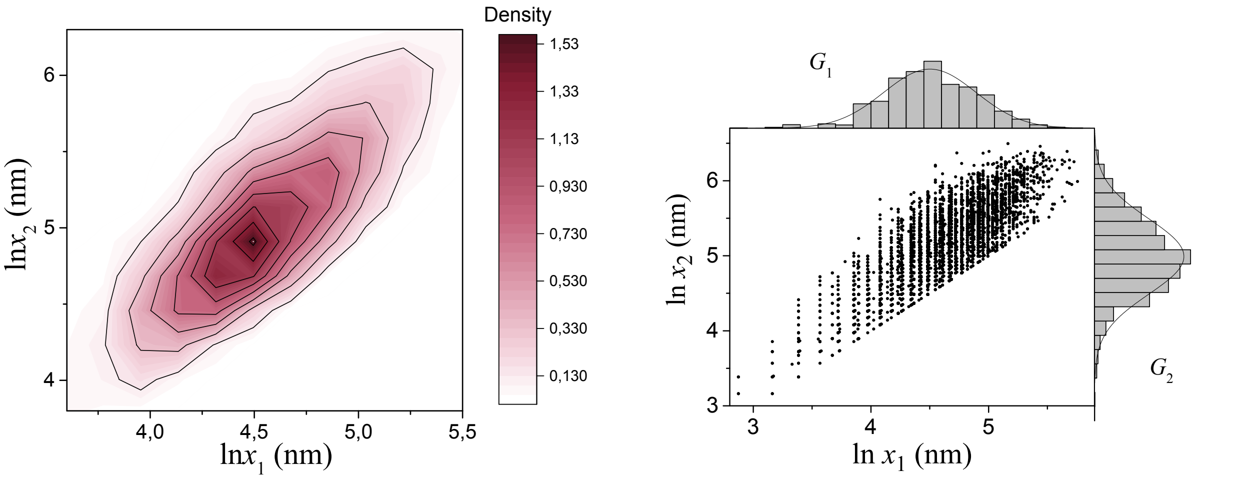

Figure S3. A possible variant of matrix isoline and two histograms G_1 and G_2 of marginal distributions.

To obtain the cumulative dependence of the crystallite area one can use the products of the approximating functions or the product of the corresponding values of the matrix $f_{i,k}$, using numerical calculations. [2]

[1] D. Ectors, F. Goetz-Neunhoeffer, J. Neubauer, J. Appl. Cryst. **2015**, 48,189.
[2] U. Frank, S.E. Wawra, L. Pflug, W. Peukert, *Part. Part. Syst. Charact.* **2019***, 36,* 1800554.
